# Examining Physical Activity Service Provision to Culturally and Linguistically Diverse (CALD) Communities in Australia: A Qualitative Evaluation

**DOI:** 10.1371/journal.pone.0062777

**Published:** 2013-04-26

**Authors:** Cristina M. Caperchione, Gregory S. Kolt, W. Kerry Mummery

**Affiliations:** 1 School of Health and Exercise Sciences, University of British Columbia, Kelowna, British Columbia, Canada; 2 School of Science and Health, University of Western Sydney, Sydney, New South Wales, Australia; 3 Faculty of Physical Education and Recreation, University of Alberta, Edmonton, Alberta, Canada; Edinburgh University, United Kingdom

## Abstract

Strong evidence exists for the role of physical activity in preventing and managing a range of chronic health conditions. A particular challenge in promoting physical activity as a health strategy exists in culturally and linguistically diverse (CALD) groups, as such groups demonstrate high risk for a range of non-communicable diseases. The aim of this research was to examine the perspective of multicultural health service providers for CALD groups with respect to the physical activity services/initiatives on offer, access barriers to these services, and ideas for future service delivery in this area. Semi-structured interviews were conducted with 15 multicultural health service providers across the capital cities of the three most populous states in Australia (New South Wales, Queensland, and Victoria), and thematic content analysis was used to examine the data. Findings indicated that the majority of physical activity initiatives were associated with organizations offering other social services for CALD communities but were greatly restrained by resources. As well, it was found that most services were not designed by taking into account specific cultural requirements for CALD communities or their cultural expectations. Common barriers identified to service uptake were classified as socio-cultural (e.g., gender, language, context of health) and environmental (e.g., transportation) in nature. These findings should be utilized when planning future physical activity and health promotion initiatives for increasing CALD participation. In particular, programs need to be culturally tailored to the specific expectations of CALD groups, addressing cultural safety and sensitivity, and should be in partnership with other organizations to extend the reach and capacity.

## Introduction

It has well been established that Australia is rich in cultural diversity. Of all countries belonging to the Organization for Economic Cooperation and Development (OECD) [1] Australia has the second largest proportion of overseas-born people in its population. Those born overseas comprise of approximately 23% of Australia’s total population, representing more than 250 different ancestries [2]. Over the next decade it is projected that the proportion of the overseas-born population, commonly referred to as culturally and linguistically diverse (CALD), will increase more rapidly than the Australian-born population [3].

With this rapidly growing diverse population Australia faces a number of population health challenges. Close examination of epidemiological data reveals particular burdens of disease in these CALD communities [4], revealing higher rates of risk factors for a number of chronic diseases in CALD populations [5], [6]. Of particular concern is the greater risk of hypertension, diabetes, and overweight/obesity, all of which are predominant risk factors to cardiovascular disease (CVD) [6], [7].

Despite the high risk of these diseases, those from CALD groups are less likely to be proactive in accessing health care or undertaking preventative health initiatives to ensure optimal health outcomes [8], [9]. It has well been documented that CALD communities, are less likely to utilize preventative health services compared to their Australian-born counterparts [10], [11], [12]. This trend is not isolated to CALD communities in Australia alone. Similar findings have been reported in the UK [13], Canada [14], New Zealand [15] and USA [16]. The reasons most commonly reported for the lower utilization of services include language issues (particularly poor English language proficiency), lack of gender segregation, a reluctance to deal with government organizations, lack of cultural awareness on the part of the health workers, a lack of knowledge/awareness of services, and differences between generic and multicultural services [3], [13], [17], [18]. Under-utilization of health services has also been associated with a fear of loss of independence, privacy and confidentiality [19]. The majority of published research has concentrated on the under-utilization of general health care services including home and community care services [20], general primary health care [21], [22], and mental health and social wellbeing services [23]. No research, however, has addressed the under-utilization of preventive health measures such as physical activity.

Engaging in regular physical activity is associated with reduced risk of cardiovascular disease and other chronic diseases [24], however those from CALD groups are less likely to access or utilize these types of programs or initiatives [25], [26]. A recent review by Horne and Tierney [27] highlighted the prevalence of this specific to the South Asian population living in both the UK and Canada, reporting that these individuals have greater rates of coronary heart disease, type 2 diabetes and vascular problems, and are less likely to be physically active compared to their Caucasian counterparts. In order to address this concern, researchers and health professionals need to be aware of why CALD populations are not accessing or utilizing these services. Recent evidence has provided preliminary insight into these issues from the perspective of CALD community members [9], [28], commonly suggesting that these services may not be appropriately adapted or tailored to be meet the cultural requirements or needs of CALD populations [29], [30], however, little work has addressed these issues from the perspective of service providers. Thus, based on the perspective of service providers, the purpose of this study was: (1) to explore the physical activity services/initiatives which are currently provided to CALD communities living in the three most populous states in Australia (New South Wales, Victoria, and Queensland); (2) to investigate service providers’ perceptions as to why these communities are not accessing these services; and (3) to explore the provision of future services to promote and support health promotion and physical activity in CALD groups throughout Australia.

## Methods

### Ethics Statement

This study was conducted in accordance with the National Health and Medical Research Council’s National Statement on Ethical Conduct in Human Research [31] and ethical approval was obtained from the Central Queensland University’s Human Research Ethics Committee (EC00158), reference number H09/02-007. Standard university guidelines of informed consent, voluntary participation, confidentiality, and anonymity were followed; Information sheets and consent forms were sent to participants prior to the scheduled interview, providing information about the project, and outlining the procedures of the interview. Information regarding voluntary participation was included, indicating that participants could withdraw from the study before, during or after the interview and up to the point of publication of the research findings, for any reason, without prejudice or consequence. Once publication has occurred, data cannot be withdrawn. Consent forms further outlined that participants’ identities would be protected at all times throughout the data collection process, analysis and publication of any research findings, indicating that any research reports or publications would be drawn from aggregated data rather than from any one participant, organization or community. Participants were asked if they would like a final report outlining the study outcomes and if so, they were required to provide an email and mailing address. Access to anonymised versions of all interview transcripts was not available to participants or any other researchers beyond the research team. Upon completion of the study, access to all raw data, including anonymised versions of the interview transcripts, remained restricted to the research team as participants only gave consent for this data to be accessed by the named research team members (CMC, GSK, WKM). Thus, data has not been and will not be deposited into a central repository and at no time will the data be made available for secondary analysis by other researchers. Participants did receive access to their own individual transcript for the purpose of reviewing for report accuracy. Prior to each interview session the principal researcher went over the information sheet and consent form once again, at which time participants were given an opportunity to ask any questions or withdraw from the study. All participants agreed to participate and written informed consent was obtained from each participant.

### Settings and Participants

This study was conducted with Multicultural Health Service Providers (MHSP) employed by government and non-government agencies/organizations throughout the capital cities of Sydney, New South Wales; Melbourne, Victoria; and Brisbane, Queensland. These cities were chosen for the reason that they have become three of the most CALD populated cities in Australia (31.7% of CALD communities residing in Sydney, 28.9% in Melbourne, and 21.7% in Brisbane) and thus service provision will not only have the greatest impact on these communities, but resources to provide services will also be the most strained in these areas [2]. Participants were recruited through an online and manual search of organizations that provided health promoting services to CALD communities throughout these three areas. Once these organizations had been identified, the principal researcher contacted each organization and was then directed to the individual responsible for the health promotion branch/section of the organization. The principal researcher provided information about the research, specifically outlining the purpose of the study and invited each multicultural health service provider (MHSP) to participate. All contacted MHSPs agreed to participate, at which time interview dates and times were scheduled.

All participants were employed by agencies/organizations which directly provided physical activity health promotion services to local CALD communities or were health promotion advocates who directly supported organizations which provided physical activity health promotion services to local CALD communities. Table 1 provides an outline of the CALD communities that are serviced by these service provision organizations. A total of 15 MHSP’s (2 men and 13 women), with a mean age of 37.4 years, participated in the interviews. Table 2 provides an outline of demographic characteristics of the service providers.

**Table 1:** CALD communities serviced by the Service Provider Organizations in each State.

New South Wales	Victoria	Queensland
Afghani	Burmese	Afghani
Albanian	Chinese	Bosnian
Bosnian	Eritreans	Burmese
Chinese	Ethiopian	Chinese
Congolese	Egyptian	Egyptian
Egyptian	Fijian	Filipino
Filipino	Filipino	Greek
Indian	Greek	Indian
Iraqi	Italian	Iraqi
Lebanese	Lebanese	Lebanese
Macedonian	Maltese	Nigerian
Maltese	Russian	Pakistani
Russian	Somali	Samoan
Serbian	Sri Lankan	*Spanish speaking
Sierra Leone	Sudenese	Sri Lankan
*Spanish speaking	Timorese	Sudanese
Sudenese	Vietnamese	Vietnamese
Vietnamese		

* Note: Spanish speaking category incorporates a small proportion of individuals from a number of Spanish speaking countries, predominately from Spain and Chile, but also including El Salvador, Honduras, Peru and Ecuador.

**Table 2:** Demographic Characteristics of the Service Providers.

Variable	Participants (N = 15)
Gender	
Female	13
Male	2
Mean Age (±SD)	37.4 (11.20)
Education	
Secondary	2
Post Secondary	13
Post Secondary Training	
Health Promotion	4
Nursing	1
Physiotherapy	2
Psychology	2
Social Work	3
Massage Therapy	1
*Health Promotion Training	15
Mean Yrs in Current Role (±SD)	6.2 (5.11)

* All participants received formal health promotion training within their workplace.

### Procedures

Qualitative methodology, involving semi-structured interviews, was employed to gather detailed information from MHSPs. In order to gather a wide range of perspectives, opinions and ideas concerning service provision, we aimed to include a varying selection of MHSPs throughout each of the three targeted areas. Semi-structured interviews were chosen because they provide the flexibility to explore experiences and attitudes, enabling the researcher to enter new areas and produce richer data [32].

Fifteen in depth, semi-structured interviews were undertaken between February 2010 and May 2010. Researchers from the project team travelled to each of the three states, undertaking the interviews at each of the MHSP’s place of employment (5 in NSW, 4 in VIC and 6 in QLD). Each interview session ranged from 30–70 minutes in length during which time MHSP’s were asked to report on established physical activity policies and services and the barriers associated with existing services, and to suggest potential strategies/services that would better meet the specific needs of CALD populations living in each area. The interview schedule was developed based on previous research addressing healthcare service provision [20], and the objectives of the current study. The schedule was guided by twelve questions, including such questions as; ‘Does your organisation provide any current physical activity or health promotion programs? If so, can you outline what programs your organisation does offer’ or ‘Why do you think CALD communities do not access some of the physical activity/health promotion programs offered by your service/organisation or other organisations? and ‘What sorts of physical activity services/opportunities would you like to see offered by your organisation or other organisations for CALD populations that would help to make them more active or health conscious? During each interview, the principal researcher acted as the moderator, guiding the discussion and providing assistance where needed, whilst the secondary researcher acted as a scribe to take notes, and was responsible for the audio recording of each session.

### Data Management and Analysis

Following each of the interview sessions the data were professionally transcribed verbatim from digital audio files. The transcripts were reviewed and corrected by the interviewers for accuracy. Participants were only given access to their own interview transcripts. Data saturation was reached by the final interview. A thematic analysis [33] was undertaken, using an inductive approach to identify, code and categories the data according to pre-determined themes. Thematic analysis is a qualitative research method which has been widely used in health promotion research [13], [34] given its capabilities to identify, analyze and report patterns within large data sets. It organizes and describes a data set in rich detail, and frequently goes beyond this and interprets various aspects of the research topic, highlighting similarities and differences, and generating unanticipated insights [33], [35].

Initially, transcribed interviews were read, with marginal remarks and memoing used to identify initial units of meaning from the text. The interviews and memos were then read and re-read, and assigned sub-themes which were categorized under predetermined higher order themes according to the study objectives and research literature. Inter-coder reliability testing was performed with another member of the research team to enhance the rigor of sub-themes. Once all coding was complete, the sub-themes were openly discussed amongst research team members to ensure that bias was minimized. Any disagreements or concerns that had arisen during the analysis were presented at this time and further discussion was carried out until consensus was reached and agreement was met. Once agreement of the sub-themes was finalized, they were then categorized into higher order themes.

## Results

### Provision of Physical Activity Programs/Initiatives

Participants acknowledged the fundamental need for programs and initiatives which promoted preventative health for CALD populations. Many of the participants outlined new programs/initiatives that are now being offered, yet they also insisted that these initiatives were long overdue and remained limited. A common trend amongst the participant responses was that the majority of the current programs where linked to or associated with external organizations offering other social services or programs specific to migrant populations. For example, one participant (an employee from a service provider which mainly focused on community social services) began a PA program for older CALD communities in conjunction with an already established program from the local YMCA. Although this partnership helped to alleviate some of the resource strain associated with developing and running new programs, participants indicated that this partnership would need to be reformatted for future program sustainability.

There was universal recognition from participants that the limited PA programs and/or health promotion initiatives currently on offer or under development were minimal and did not necessarily meet the needs of the intended CALD communities. Participants clarified that limited resources, including financial resources and trained staff, were significant contributors to the limited programs offered to CALD communities. They indicated that initial funds and staff are first allocated to “priority” services such as migration services, family welfare services, and employment services, and thus little money is left to subsidies preventative health services such as physical activity programs or initiatives. Moreover, the programs/initiatives that do exist are not specifically tailored to meet the cultural needs of each CALD community but, rather, are mainly minor adjustments to mainstreams activities. This is illustrated by the following comment.

*Personally I’d like to see more of a proactive approach to a lot of things, including I guess physical activity, rather than a reactive approach, which is often the way things go, that would allow for really serious consideration to cultural factors at that very early planning stage. (male, 32 years, Sydney)*

### Barriers to Accessing and Utilizing Programs/Initiatives

In addition to the challenges surrounding the provision of PA programs or health promotion initiatives for CALD communities, or lack thereof, participants also highlighted common barriers they have witnessed concerning access to or utilization of health promotion programs/initiatives for CALD communities. Most prevalent were the socio-cultural barriers, in particular, misconceptualisation of preventative health and preventative health measures, language difficulties, and inappropriate nature of activities (e.g. gender issues). The majority of interview participants indicated that many CALD communities are unclear as to what constitutes preventative health or why we should engage in it. For these communities, illness and disease is regarded as a natural part of life, that being diagnosed with chronic disease or other disease complications is what is intended and the way life is supposed to be. Individuals from CALD groups do not understand the health benefits associated with engaging in preventative health measures like PA, they cannot conceptualize the preventative nature of such activities. The following resonates many of the participant responses;

*The cultural expectations are different in different communities, and I think generally we’ve seen from our experience, that those in ethnic communities are more likely to accept things like frailty and ill health as a natural part of ageing than perhaps an Anglo background person might. So they’re less likely to go for help because they’ll just accept it. (female, 27 years, Brisbane)*

All participants agreed that the idea of prevention or early intervention is not understood by many CALD communities, many would much rather go to a doctor, have medicine prescribed to them and return home to continue on with their daily routine. When they get sick again, the process repeats itself, they head back to the doctor for another ‘quick fix’ as this was common practice in their country of origin.

As alluded to in the preceding response by one participant, language is also regarded as a fundamental barrier to accessing, utilizing, and returning to PA programs or health promotion initiatives. Many participants indicated that individuals from CALD communities rarely access these initiatives because most of the time advertising and marketing messages are in English, and thus these individuals are not even aware that the programs exist.

*There is certainly a language barrier and understanding really what a certain program is about. They just start thinking that this must not be that important……(female, 39 years, Sydney)*

If they are aware and decide to participate, many are reluctant to return because the program is led by an English-only speaking facilitator/instructor and the activities that are undertaken are often foreign to these CALD communities. Thus, the inability to understand instructions and to communicate and engage with the facilitator/instructor is compounded by the unfamiliar, and sometimes inappropriate, physical activities that are encouraged to be performed. As one respondent put it:

*The barrier for CALD communities becomes when the information is provided in a way that isn’t appropriate to them. There are lots of people who have language barriers in terms of comprehension…… issues to do with health literacy and people’s understanding of the importance of physical activity. (female, 40 years, Melbourne)*

Participants clearly indicated that many of the services or programs on offer do not meet the specific needs of the target group, further highlighting that many of the physical activities promoted are recognized as inappropriate for some CALD communities due to the environment in which they are carried out, the instructors who lead them, and/or the overall nature of the activity itself. A common example described by a large proportion of the participants was the programs being mixed gender, pointing out that these programs do not take into account the religious and cultural differences of some communities in which males and females cannot participate in the same program at the same time. In an attempt to resolve such issues, one response, which follows a common theme amongst all participants, included:

*What tends to happen I think, is that organizations have a model that they kind of apply to all communities and occasionally may alter it, you know, have a bilingual facilitator, for example to address language barriers. But in its inception the model doesn’t take into account cultural factors and cultural differences. (female, 40 years, Melbourne)*

Socioeconomic and environment issues have also been observed as significant barriers to accessing and utilizing PA programs or health promotion initiatives. Of particular concern, in terms of socioeconomic barriers, are the expenses associated with the actual programs and childcare services needed in order to participate in the programs. Participants were in agreement that the majority of CALD communities would focus their financial resources on daily necessities rather than health promotion programs. As one respondent highlighted:

*I think that these individuals are too busy focusing on housing, clothing, family interests, schooling for their children, food and employment rather than worry about community-based activity programs and how they could access them. (female, 39 years, Brisbane)*

Lack of transport was recognized as the most prominent environmental barrier for not accessing or utilizing PA programs or health promotion initiatives. This is based not only on the expenses associated with transport, but also the physical ability to get to and from destinations,

*….they can’t get to it because they haven’t got transport, they haven’t got the family networks to actually take them to where they need to go. (male, 32 years, Sydney)*

In response to some of the barriers mentioned above, participants also proposed future service and/or program provision ideas, as well as outlined particular strategic adjustments in which community organizations and service providers should take into account when developing and delivering services and/or programs for local CALD communities.

### Considerations for the Future Provision of Physical Activity Programs/Initiatives

Unanimously, participants indicated that services and programs offered to CALD communities throughout Australia need to be tailored to the needs and ‘wants’ of each particular community.

*We need to navigate the different ways different cultures do things…It is important to understand all of the different cultural norms in order to be able to access them. (female, 50 years, Sydney).*

These tailored programs need to go beyond the basic translation of language and design of culturally appropriate images, but must also consider other institutions within each diverse culture, such as religion, gender, family, education, migration experiences, and the possible physical and mental stress and trauma that many of these CALD communities have faced in their past, as well as the challenges they continue to face at the present time. Furthermore, participants indicated that in order to successfully tailor these programs, individuals from each CALD community need to be involved in the design, development, and delivery of these programs. A common response from participants included;

*What we need to do is sit down with communities and find out what they want to do, what their priority is. We can’t say ‘oh walking is good, walking is cheap, here do some walking’, without it coming from them. We have to get them to start thinking about their own ideas and from there work together and come up with how we can help them start doing what they think is important to them and what they would like to do.(female, 34 years, Melbourne).*

In support of culturally tailored programs, many participants further indicated that making these programs and/or services more accessible to the specific communities should also be considered.

*Taking the programs to them, making it very accessible in terms of location, availability of childcare, cost, you know all of those thing. (female, 50 years, Sydney)*

And

*We can’t just hold ourselves up here and say we’ve got this group here, try it. We need to go to them, access some of their festivals and things to be more integrated with them, be more visible to them. (female, 22 years, Brisbane).*

Additionally, one participant went so far to suggest the development of *“one stop shops”* (*female, 32 years, Brisbane)* in order meet a number of the culturally specific needs of the groups, rather than physical activity alone.

Commonly stated by participants was the need for community partnerships and the cross promotion of programs and initiatives within these partnerships. Participants indicated that in developing relationships with other organizations, both traditional (e.g., YMCA, community health departments, Sport and Recreation) and non-traditional (e.g., migration resources centers, learning/language centers, social development agencies) health promotion organizations, both parties could share equipment, facilities, resources, and staff, as well as build greater awareness and increase recruitment across many communities.

## Discussion and Conclusions

In this study Multicultural Health Service Providers employed by government and non-government agencies/organizations throughout three Australian states shared their beliefs, opinions, and ideas regarding the current physical activity services/initiatives being provided, highlighted the barriers they believe CALD communities face in trying to access and utilize these services/initiatives, and outlined considerations for future services/initiatives. In terms of the current services/initiatives, participants indicated that despite the services/initiatives that are being offered, there is a fundamental need for more. In order to offer more, current services/initiatives need to extend their reach by partnering with other external organizations that also provide services to these CALD communities. Collaborative partnerships concerning health and preventive medicine is not a new phenomenon, and has been shown to improve conditions and outcomes related to health and well being of entire communities [36], [37]. Such partnerships have the ability to integrate and share a number of organizational elements, including resources, facilities, decision-making, and program organization [36], [38]. Moreover, these partnerships encourage community engagement, resulting in greater sustainability of initiatives that promote and maintain widespread health behaviors [36], [39]. Going beyond previous research and extending these partnerships to non-traditional health providers may be a way to overcoming some of the barriers CALD communities face in accessing and utilizing physical activity services [40], [41]. Developing collaborative partnerships with organizations that already provide other services to CALD communities, such as language centers, migration resource centers, and religious institutions, could assist with issues concerning staffing, facilities, equipment, and cultural insensitivity. In addition, utilizing non-health related government and non-government organizations provides an alternative avenue for recruitment [37].

On the basis of our findings, it is apparent that although some services/initiatives existed, these where not necessarily tailored to meet the unique cultural characteristics of each diverse CALD community. Developing programs, resources, and providing cultural competence and sensitivity training for facilitators, has some merit with regards to program delivery [28], [40], [42], however, a greater level of cultural awareness is required. It has been suggested that greater attention be paid to the design, delivery and implementation of the service/initiative, proposing that community-based ‘champions’ or ‘navigators’ should be involved in the development of the program from conception to delivery. As members of a particular CALD community, these ‘champions’ or ‘navigators’ could gain access into marginalized communities in a more efficient manner, and promote greater uptake of physical activity strategies, as well as build more robust community capacity in the long term [33], [43].

Misconceptualisation of preventative health and preventative health measures, language difficulties, inappropriate nature of activities, and social isolation were recognized by participants as predominant socio-cultural barriers faced by CALD communities when attempting to access or utilize health promotion services/initiatives. Although it could be argued that these barriers are distinct, from a service provision perspective these barriers are not necessarily independent of one another but are integrated around the cultural competence of those providing the services/initiatives. Successful access and utilization of preventive health services amongst CALD communities is less apparent due to lack of knowledge among providers about what constitutes effective culturally appropriate services [11], [44]. In order to alleviate some of the misconceptions surrounding preventive health care, organizations providing services need to design and develop culturally safe services/initiatives, where there is no distress on a person’s identity which may be caused by the fact that the service delivery methods or processes are unfamiliar to the person’s culture [45]. These could include addressing language difficulties associated with utilizing services and developing culturally appropriate initiatives to fit the needs of the CALD communities. For instance, CALD groups of Muslim faith require gender segregation in order to participate in physical activity, thus culturally appropriate adjustments need to be made to activities, activity facilities, and activity session times, creating suitable woman-only alternatives [13], [28]. Henderson et al., [12] indicated that in order to provide culturally safe services, it is necessary to embrace the differences that are inherent across cultures, taking into consideration that something that may work for one CALD group, may not necessarily work for another CALD group. Service providers must be willing to understand and accept all aspects of each diverse culture and adjust services/initiatives accordingly. In addition, services/initiatives are more likely to become culturally safe and culturally appropriate through the use of cultural knowledge, culturally appropriate processes, and continued cultural sensitivity [41], [46], [47]. Anderson et al. [46] suggested that in order to address knowledge, appropriateness, and sensitivity, organizations must promote a service that; 1) includes a culturally diverse staff that reflects the communities served; 2) provides translators who speak the clients language/s; 3) provides training for service providers/facilitators about the culture and language of the people they serve; 4) includes signage and instructional literature in the clients’ language and consistent with their cultural norms, and 5) provides culturally specific environments/settings for delivery. As outlined, providing culturally competent services (inclusive of the factors highlighted above) is necessary in overcoming access and utilization barriers as it has the potential to increase efficiency of service as well as enable CALD people to approach services without fear and uncertainty, whilst respecting their own health beliefs.

For many CALD groups, the health and welfare of their family is their number one priority, thus socioeconomic barriers become present in view of the fact that all financial resources they have are attached to “daily life necessities” (e.g., food, clothes, school, childcare, transport) with usually no money left for recreational or leisure type activities. In an attempt to address these economic struggles, service providers could help to alleviate some of these concerns. For instance, research has consistently recognized childcare (the expense associated with it) as one of the most cited barriers to physical activity participation for adults, in particular CALD women [28], [48], [49], suggesting that service providers should consider offering free or highly subsidized childcare at their facility as a way to address this barrier [49], [50]. The burden of getting children to and from a childcare facility, and then paying the fee for the service would be alleviated and allow CALD clients more opportunities to participate in the organized program/initiative.

Although transportation has traditionally been defined as an environmental barrier, our findings recognize it as both an environmental and socioeconomic barrier, given the actual expense associated with transporting to and from a geographical location. In accordance with what was suggested to improve the childcare barrier, there has also been some discussion around service provider organizations assisting with transportation to and from the activity or program venue by subsidizing public transport [41] or potentially providing private transport for activity participants, similar to hotel or airport shuttles. Environmentally, transport becomes a barrier for CALD communities as many of them are unfamiliar with the process of public transport and are fearful of taking public transport. Service providers could provide some assistance with this barrier by improving their knowledge around using public transportation. For example, during the initial stages of the activity program facilitators could organize a walk which involved taking the bus to and from a desired walking location. This would give participants hand on knowledge about the public transport process as well as provide an opportunity for physical activity and social interaction.

It is clear that the future design and development of physical activity programs/initiatives must be centered on some of the key aspects discussed earlier. In particular, developing and fostering collaborative partnerships with traditional and non-traditional organizations, tailoring programs to target the specific cultural needs of each particular CALD group, and increasing accessibility by alleviating the barriers to PA is central to the future provision and sustainability of PA programs/initiatives. For example, in the case of accessibility our findings indicated that “taking the programs to them” is a service approach that would alleviate many of the barriers faced by these groups. Parallel to the work undertaken in the United Kingdom [51], local health service providers could set up a ‘one stop shop’ for both primary and secondary health promotion initiatives (with links to other common migrant services such as housing and language services) in the geographical locations where CALD communities reside. Referred to as “health action zones” [51], these centers/facilities would provide culturally tailored primary health care services, health promotion resources, and preventative health promotion programs/initiatives that are easily accessible and that provide an environment that is culturally sensitive to the needs of the targeted CALD group. Although this strategy may elicit worry concerning social isolation, it is important to note that these ‘zones’ are not meant to segregate individuals, but rather this approach will provide a culturally safe environment for CALD groups to access PA programs/initiatives, as well as master skills (e.g., physical activity skills, language skills, etc) and further cultivate their confidence and self-efficacy so they feel safe and comfortable with potentially transitioning beyond their own cultural environment.

### Strengths, Limitations and Future Recommendations

This research makes a significant contribution to the literature as it explores the perceptions of CALD services providers regarding PA service provision, a research area which has most commonly reported on CALD community members accessing these services, rather than those who actually offer or provide these services. As such, this work provides a preliminary outlook, from the service provider’s perspective, as to what programs/initiatives are currently provided to CALD communities, why these communities are not accessing these services, and what are some of the strategies that could be used to promote and support health promotion and physical activity services/programs/initiative in CALD groups throughout Australia. Furthermore, the outcomes from this study do provide important information that would be attractive to an international audience given the similar migration patterns found in other western countries such as Canada [52], US [53], and the UK [54]. Although specific details cannot be drawn from these data given the diversity of many CALD communities worldwide, this preliminary outlook, which highlights the importance of cultural safety, cultural sensitivity and cultural competence within service provision, does provide a starting point for service providers to design, develop, and/or alter new and existing programs to better accommodate the cultural requirements and personal needs of these communities.

Despite the valuable contributions of this work, this study is specific to service provision in three Australian states (NSW, Queensland, Victoria) thus making it difficult to generalize these findings throughout all organizations which provide services to CALD communities throughout all of Australia. Although, it has been documented that the capital cities and surrounding areas of these states have the greatest porportion of CALD populations [2], and thus the greatest need for services, there are other areas in South Australia, Western Australia and particular rural areas of Australia which are witnessing a rapid growth in CALD populations through international and inter-state migration. Future research should focus on large-representative sampling, throughout all states in Australia, to clearly understand the service provision needs of both old and new CALD communities, in addition to establishing a national profile regarding PA service provision for CALD communities.

This study specifically addressed service provision from the perspective of community level, government and non-government service providers, not taking into account the role that health care professionals may play in the process of service provision. Although utilization of healthcare is particularly low in migrant populations [20], [21], [22], [23], those who do seek primary and secondary healthcare will usually first access it from a local general medical practitioner (GP) and allied health professionals, as they are often referred to as the “gatekeepers” of health for immigrant populations [55]. This preliminary visit is an ideal time for GPs and other allied health professionals to advocate for preventative health care, in particular providing information about physical activity, highlighting specific local services and encouraging participation of these services. Examining the influence that health professionals may have on the utilization of services for CALD groups is a research area that warrants future attention.

### Conclusions

The outcomes of this exploratory research outline a number of implications that should be considered for practice, policy and research. Developing and fostering formal partnerships with non-traditional organizations which service CALD communities (e.g., language centers, migration service centers, social welfare organizations) is necessary as these reciprocal relationships have the potential to provide support and assistance in terms of shared resources, building greater awareness of the services within the community, and recruiting community members into the services or initiatives. Greater initiative must also be taken by organizations to mandate cultural specific training for facilitators of these services/initiatives to ensure that these service providers are equipped with a sufficient level of cultural competency to accommodate the specific cultural needs of these CALD communities. These training sessions must focus specifically on the diversity within each CALD community and provide knowledge concerning cultural safety and sensitivity. Understanding these aspects and how they influence each CALD community differently will assist service providers in addressing the barriers associated with the utilization of these services as well as provide them with the knowledge to provide the most appropriate service. In looking forward, there are many areas which warrant further investigation concerning physical activity service provision for CALD communities. A closer examination of other external variables, such as the influence of health professionals in encouraging utilization of physical activity service provision, would be a valuable and logical extension to this research and would make a significant contribution to the existing literature.

## References

[1] Dumont JC, Lemaitre G (2005) Counting immigrants and expatriates in OECD countries: A new prespective.: Department of Economic and Social Affairs. 25 OECD Social, Employment and Migration working papers 25 OECD Social, Employment and Migration working papers.

[2] Australian Bureau of Statistics (2006) 2006 Census of Population and Housing. Canberra:ACT. Cat. no.2914.0 Cat. no.2914.0.

[3] Warburton J, Bartlett H, Rao V (2009) Ageing and cultural diversity: Policy and practice issues. Aust Soci Work 62: 168–185.

[4] Adily A, Ward J (2005) Improving health among culturally diverse subgroups: an exploration of trade-offs and viewpoints among a regional population health workforce. Health Promot J Austr 16: 207–212. 16375036 10.1071/he05207

[5] Davidson PM, Daly J, Hancock K, Jackson D (2003) Australian women and heart disease: trends, epidemiological perspectives and the need for a culturally competent research agenda. Contemp Nurse 16: 62–73. 14994897 10.5172/conu.16.1-2.62

[6] Johnson PA, Fulp RS (2002) Racial and ethnic disparities in coronary heart disease in women: prevention, treatment, and needed interventions. Womens Health Issues 12: 252–271. 12225688 10.1016/s1049-3867(02)00148-2

[7] Torpy JM, Lynm C, Glass RM (2003) JAMA patient page. Risk factors for heart disease. JAMA 290: 980. 12928476 10.1001/jama.290.7.980

[8] Gettleman L, Winkleby MA (2000) Using focus groups to develop a heart disease prevention program for ethnically diverse, low-income women. J Community Health 25: 439–453. 11071226 10.1023/a:1005155329922

[9] Caperchione CM, Kolt GS, Mummery WK (2009) Physical activity in culturally and linguistically diverse migrant groups to Western society: a review of barriers, enablers and experiences. Sports Med 39: 167–177. 19290674 10.2165/00007256-200939030-00001

[10] Marquez DX, McAuley E (2006) Gender and acculturation influences on physical activity in Latino adults. Ann Behav Med 31: 138–144. 16542128 10.1207/s15324796abm3102_5

[11] Comino EJ, Silove D, Manicavasagar V, Harris E, Harris MF (2001) Agreement in symptoms of anxiety and depression between patients and GPs: the influence of ethnicity. Fam Pract 18: 71–77. 11145632 10.1093/fampra/18.1.71

[12] Henderson S, Kendall E, See L (2011) The effectiveness of culturally appropriate interventions to manage or prevent chronic disease in culturally and linguistically diverse communities: a systematic literature review. Health Soc Care Community 19: 225–249. 21208326 10.1111/j.1365-2524.2010.00972.x

[13] Jepson R, Harris FM, Bowes A, Robertson R, Avan G, et al (2012) Physical activity in South Asians: an in-depth qualitative study to explore motivations and facilitators. PLoS One 7: e45333. 23071511 10.1371/journal.pone.0045333 PMC3468573

[14] Oliffe JL, Grewal S, Bottorff JL, Hislop TG, Phillips M, et al.. (2009) Physical activity among senior South Asian Canadian immigrant men: Connecting masculinities and culture. Crit Publ Health 19 383–398.

[15] Kolt GS, Paterson JE, Cheung VYM (2006) Barriers to physical activity participation in older Tongan adults living in New Zealand. Australas J Ageing 25: 119–125.

[16] Marshall SJ, Jones DA, Ainsworth BE, Reis JP, Levy SS, et al (2007) Race/ethnicity, social class, and leisure-time physical inactivity. Med Sci Sports Exerc 39: 44–51. 17218883 10.1249/01.mss.0000239401.16381.37

[17] Fuller J, Ballantyne A (2000) Immigrants and equitable health-care delivery in rural areas. Aust J Rural Health 8: 189–193. 11894283 10.1046/j.1440-1584.2000.00270.x

[18] Fennelly K (2007) The “healthy migrant” effect. Minn Med 90: 51–53. 17432759

[19] Khan N (1994) Health services in a multicultural Queensland: A silent crisis. Brisbane: Ethnic Communities Council of Queensland.

[20] Ward B, Ellis J, Anderson K (2005) Barriers to the provision of home and community care services to culturally and linguistically diverse populations in rural Australia. Austr J Primary Health 11: 147–155.

[21] Asanin J, Wilson K (2008) “I spent nine years looking for a doctor”: exploring access to health care among immigrants in Mississauga, Ontario, Canada. Soc Sci Med 66: 1271–1283. 18194831 10.1016/j.socscimed.2007.11.043

[22] Sundquist J (2001) Migration, equality and access to health care services. J Epidemiol Community Health 55: 691–692. 11553650 10.1136/jech.55.10.691 PMC1731775

[23] Wynaden D, Chapman R, Orb A, McGowan S, Zeeman Z, et al (2005) Factors that influence Asian communities’ access to mental health care. Int J Ment Health Nurs 14: 88–95. 15896255 10.1111/j.1440-0979.2005.00364.x

[24] Bauman AE (2004) Updating the evidence that physical activity is good for health: an epidemiological review 2000–2003. J Sci Med Sport 7: 6–19. 15214597 10.1016/s1440-2440(04)80273-1

[25] Australian Bureau of Statistics (2006) Migrants and participation in sport and physical activity 2006. Canberra, ACT: National Centre for Culture and Recreation Statistics.

[26] Walseth K, Fasting K (2003) Islam’s view on physical activity and sport: Egyptian women interpreting Islam. Int Rev Socio Sport 38: 45–60.

[27] Horne M, Tierney S (2012) What are the barriers and facilitators to exercise and physical activity uptake and adherence among South Asian older adults: a systematic review of qualitative studies. Prev Med 55: 276–284. 22846506 10.1016/j.ypmed.2012.07.016

[28] Caperchione CM, Kolt GS, Tennent R, Mummery WK (2011) Physical activity behaviours of Culturally and Linguistically Diverse (CALD) women living in Australia: a qualitative study of socio-cultural influences. BMC Public Health 11: 26. 21223595 10.1186/1471-2458-11-26 PMC3091537

[29] Liu JJ, Davidson E, Bhopal RS, White M, Johnson MRD, et al (2012) Adapting health promotion interventions to meet the needs of ethnic minority groups: mix-methods evidence synthesis. Health Technol Assess 16: 1–468. 10.3310/hta16440 PMC4780992 23158845

[30] Babakus WS, Thompson JL (2012) Physical activity among South Asian women: a systematic, mixed-methods review. Int J Behav Nutr Phys Act 9: 150. 23256686 10.1186/1479-5868-9-150 PMC3542106

[31] National Health and Medical Research Council (2007) National Health and Medical Research Council’s National Statement on Ethical Conduct in Human Research. Canberra: Australian Government: National Health and Medical Research Council.

[32] Pope C, van Royen P, Baker R (2002) Qualitative methods in research on healthcare quality. Qual Saf Health Care 11: 148–152. 12448807 10.1136/qhc.11.2.148 PMC1743608

[33] Guest G (2012) Applied thematic analysis. Thousand Oaks, California: Sage. 320 p.

[34] Lawton J, Ahmad N, Hanna L, Douglas M, Hallowell N (2006) ‘I can’t do any serious exercise’: barriers to physical activity amongst people of Pakistani and Indian origin with Type 2 diabetes. Health Educ Res 21: 43–54. 15955792 10.1093/her/cyh042

[35] Braun V, Clarke V (2006) Using thematic analysis in psychology. Qual Res Psychol 3: 77–101.

[36] Roussos ST, Fawcett SB (2000) A review of collaborative partnerships as a strategy for improving community health. Annu Rev Public Health 21: 369–402. 10884958 10.1146/annurev.publhealth.21.1.369

[37] Van Duyn MS, McCrae T, Wingrove B, Henderson KM, Boyd JK, et al (2007) Adapting evidence based strategies to increase physical activity among African Americans, Hispanics, Hmong, and Native Hawaiians: A social matketing approach. Prev Chronic Dis 4: 1–11. PMC2099267 17875246

[38] Yancey AK, Ory MG, Davis SM (2006) Dissemination of physical activity promotion interventions in underserved populations. Am J Prev Med 31: S82–91. 16979472 10.1016/j.amepre.2006.06.020

[39] Yancey AK, Miles O, Jordan A (1999) Organizational characteristics facilitating initiation and institutionalization of physical activity programs in a multi-ethnic, urban community. J Health Educ 30: S44–S51.

[40] Belza B, Walwick J, Shiu-Thornton S, Schwartz S, Taylor M, et al (2004) Older adult perspectives on physical activity and exercise: voices from multiple cultures. Prev Chronic Dis 1: A09. PMC1277949 15670441

[41] Queensland Health (2010) Engaging culturally and linguistically diverse (CALD) Queenslanders in physical activity: Findings of the CALD Physical Activity Mapping Project. Brisbane: Division of the Chief Health Officer, Queensland Health.

[42] Kreuter MW, Lukwago SN, Bucholtz RD, Clark EM, Sanders-Thompson V (2003) Achieving cultural appropriateness in health promotion programs: targeted and tailored approaches. Health Educ Behav 30: 133–146. 12693519 10.1177/1090198102251021

[43] Brach C, Fraser I (2000) Can cultural competency reduce racial and ethnic health disparities? A review and conceptual model. Med Care Res Rev 57 Suppl 1181–217. 11092163 10.1177/1077558700057001S09 PMC5091811

[44] Rao DV, Warburton J, Bartlett H (2006) Health and social needs of older Australians from culturally and linguistically diverse backgrounds: issues and implications. Australas J Ageing 25: 174–179.

[45] Ramsden I (1990) Cultural Safety. New Zeal J Nurs 83: 18–19. 2267106

[46] Anderson LM, Scrimshaw SC, Fullilove MT, Fielding JE, Normand J (2003) Culturally competent healthcare systems. A systematic review. Am J Prev Med 24: 68–79. 12668199 10.1016/s0749-3797(02)00657-8

[47] Majumdar B, Browne G, Roberts J, Carpio B (2004) Effects of cultural sensitivity training on health care provider attitudes and patient outcomes. J Nurs Scholar 36: 161–166. 10.1111/j.1547-5069.2004.04029.x 15227764

[48] Guerin PB, Elmi FH, Corrigan C (2007) Body composition and cardiorespiratory fitness among refugee Somali women living in New Zealand. J Immigr Minor Health 9: 191–196. 17252194 10.1007/s10903-006-9030-x

[49] Cortis N, Sawrikar P, Muir K (2007) Participation in sport and recreation by culturally and linguistically diverse women. Canberra, ACT: Australian Government Office for Women Department of Families, Community Services and Indigenous Affairs.

[50] Guerin PB, Diiriye RO, Corrigan C, Guerin B (2003) Physical activity programs for refugee Somali women: working out in a new country. Womens Health 38: 83–99. 10.1300/J013v38n01_06 14535608

[51] Barnes M, Bauld L, Benzeval M (2005) Health Action Zones: Partnerships for Health Equity. London: Routledge.

[52] Statistics Canada (2006) 2006 Census: Immigration in Canada: A portrait of the foreign-born population. Ottawa: Statistics Canada.

[53] Lee J, Rytina N (2009) Naturalizations in the United States:2008. Washington: Office of Immigration Statistics.

[54] Somerville W, Sumption M (2009) Immigration in the United Kingdom: The recession and beyond. Washington: Migration Policy Institute.

[55] Sole-Auro A, Guillen M, Crimmins EM (2009) Health care utilization among immigrants and native-born poulations in 11 European countries. Results from the Survey of Health, Ageing and Retirement in Europe. Barcelona: Research Institute of Applied Economics. 1–29 p.

